# The Risk of Foot and Mouth Disease Transmission Posed by Public Access to the Countryside During an Outbreak

**DOI:** 10.3389/fvets.2019.00381

**Published:** 2019-11-05

**Authors:** Harriet Auty, Dominic Mellor, George Gunn, Lisa A. Boden

**Affiliations:** ^1^Epidemiology Research Unit, Scotland's Rural College, Inverness, United Kingdom; ^2^School of Veterinary Medicine, College of Medical, Veterinary and Life Sciences, University of Glasgow, Glasgow, United Kingdom; ^3^The Global Academy of Agriculture and Food Security, The Royal (Dick) School of Veterinary Studies, The Roslin Institute, Midlothian, United Kingdom

**Keywords:** foot and mouth disease, risk assessment, policy, rural access, transmission, fomites

## Abstract

During the 2001 UK FMD outbreak, local authorities restricted rural access to try to prevent further disease spread by people and animals, which had major socio-economic consequences for rural communities. This study describes the results of qualitative veterinary risk assessments to assess the likelihood of different recreational activities causing new outbreaks of foot and mouth disease, as part of contingency planning for future outbreaks. For most activities, the likelihood of causing new outbreaks of foot and mouth disease is considered to vary from very low to medium depending on the control zone (which is based on distance to the nearest infected premises), assuming compliance with specified mitigation strategies. The likelihood of new outbreaks associated with hunting, shooting, stalking, and equestrian activities is considered to be greater. There are areas of significant uncertainty associated with data paucity, particularly regarding the likelihood of transmission via fomites. This study provides scientific evidence to underpin refinement of rural access management plans and inform decision-making in future disease outbreaks.

## Introduction

Highly contagious diseases of livestock have the potential for significant impact, not only on the agricultural industry but also the wider economy and on society. Foot and mouth disease virus (FMDV) is both easily transmissible and able to persist in the environment (1, 2), meaning that control strategies must aim to prevent transmission via fomites as well as direct contact. Therefore, foot and mouth disease (FMD) has the potential for substantial societal impact; control strategies rely not only on mandatory slaughter of infected and in-contact animals and restrictions on movement and trade of susceptible livestock species (3, 4), but may also require restrictions on the activities of non-susceptible animals and people, who may transmit the virus mechanically.

During the 2001 UK foot and mouth disease outbreak, UK local authorities took a precautionary approach to disease control and used blanket bans to close all footpaths, even in uninfected areas, to try to prevent further disease spread by people and animals. These measures had major socio-economic consequences for rural communities (5). The tourism sector suffered the greatest financial impact and is estimated to have lost around £3bn due to the outbreak, due in large part to the perception that “the countryside was closed” (6, 7). Post-outbreak reports highlighted the need for more research on the likely efficacy of biosecurity measures such as footpath closures, and more transparent risk-based decision making, particularly regarding rural access (5, 7).

Although the exceptional scale of the 2001 outbreak in UK undoubtedly exacerbated the issues of rural access (8, 9), the role of people accessing to the countryside in contributing to onward transmission of FMD remains an important question that has not been addressed in Scotland or in other countries that are FMD-free. In light of this, a suite of veterinary risk assessments (VRAs) were developed to consider the risk of disease spread associated with recreational access to the countryside. Here, we describe the risk assessments and conclusions as well as highlighting key assumptions and knowledge gaps.

## Materials and Methods

Risk questions were developed for each of 12 countryside activities: walking, cycling, canoeing, fishing, horse riding, staging equestrian events, staging a horse racing meet, staging other events on agricultural land, staging sporting events such as running competitions or triathlons, drag hunting, stalking deer, and shooting birds. For each activity, the risk question took the form “What are the risks of causing new outbreaks of foot and mouth disease (FMD) by walking, and other similar activities such as dog walking, and climbing?”

The risk assessments followed a standard approach, considering (i) hazard identification; (ii) risk pathway; (iii) legislation, definitions and assumptions; (iv) release and exposure assessment; (v) consequence assessment; and (vi) overall likelihood levels and risk management options. A qualitative approach was chosen over a quantitative approach after careful consideration of the paucity of data on which to base a quantitative assessment. Definitions of qualitative likelihood levels used were derived from those published by the World Organization for Animal Health (OIE) and adopted by the UK Department for Environment, Food and Rural Affairs (DEFRA) (10, 11) and are presented in Table 1.

**Table 1:** Definitions of qualitative likelihood estimate levels.

**Likelihood level**	**Description**
Negligible	So rare that it does not merit consideration
Very low	Very rare but cannot be excluded
Low	Rare but could occur
Medium	Occurs regularly
High	Occurs very often
Very High	Events occur almost certainly

A risk pathway was developed for each activity that identified the steps involved in release of and exposure to FMDV. A review of the available literature was used to identify all relevant factors which are likely to influence these steps. A search of the scientific literature published in peer-reviewed journals was done using the following search terms: “foot and mouth disease” and: “wildlife”; “transmission”; “fomites”; “environment”; “survival.” Important references were also identified in key review papers (12–15). In addition, previous risk assessments including those produced during the 2001 FMD outbreak in UK (9), were used to inform the exposure and release assessments. On collation of risk factors, key knowledge gaps or areas of uncertainty were identified for each step in the pathway. Likelihood estimates for each step were developed based on the information available. Likelihood estimates assumed compliance with standard statutory measures in place during an FMD outbreak but did not take into account any additional mitigation measures specific to the activities in question. In reality it is unlikely that these activities would be permitted in the absence of additional mitigation measures aimed to reduce the risk of onward disease transmission. Relevant specific mitigation measures were identified in consultation with the Animal and Plant Health Agency (responsible for implementing disease control) and additional likelihood estimates were provided for each step assuming these mitigation measures were in place. Compliance was assumed, although areas of particular concern for non-compliance were highlighted in the risk assessment. The consequences of a new outbreak as a result of the risk pathway were considered, and final risk levels based on a combination of the likelihood of exposure and release and the severity of the consequences (11). The VRAs were reviewed by the Scottish Government and the UK National Experts Group on Foot and Mouth Disease.

## Results

Individual VRAs for each activity can be seen at https://www.gov.scot/publications/foot-and-mouth-disease-veterinary-risk-assessments-vras/.

### Hazard Identification

The hazard is FMD virus. There are seven serotypes of FMD virus: O, A, C, SAT1, SAT2, SAT3, and Asia 1. Different serotypes (and different strains within each serotype) have different characteristics, including variation in host species susceptibility, length of incubation period, ease of detecting clinical signs and transmission (16–19). Much research is based on the UK 2001 outbreak, which was caused by serotype O, strain PanAsia (20). However, future outbreaks may involve other serotypes/strains and therefore present different epidemiological situations.

The specific risk is that attending, conducting or staging leisure activities in the countryside during an FMD outbreak may involve people or associated fomites that have been, or come into, contact with FMDV and with susceptible livestock, leading to FMD spread via people or other fomites to cause further disease outbreaks.

### Risk Pathway

**Figure 1:**
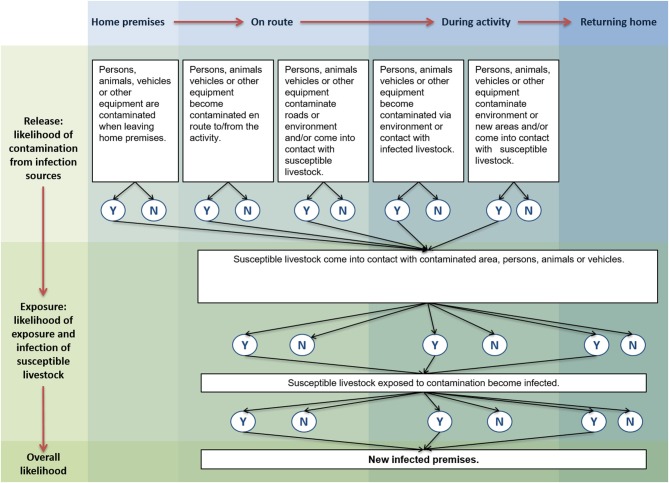
Summary risk pathway. Individual risk pathways were developed for each of 12 activities that require access to the countryside. This summary pathway illustrates the common steps in the risk pathway from release of FMDV to exposure of susceptible livestock.

Risk pathways were developed for each activity comprising release and exposure assessments. A summary risk pathway is shown in Figure 1. Release included persons, animals, vehicles or other equipment (i) already contaminated on leaving the home premises; (ii) becoming contaminated on the way to or from the activity, contaminating roads or the environment; (iii) coming into contact with susceptible livestock whilst on the way to or from the activity; (iv) becoming contaminated via the environment or through contact with infected livestock during the activity; and (v) contaminating the environment, moving contamination to new areas, or coming into contact with susceptible livestock during the activity. Exposure included susceptible livestock being exposed and ultimately infected, through coming into contact with contaminated areas, persons, animals or vehicles, and could occur on return to the home premises, whilst traveling to or from the activity, or whilst doing the activity.

### Legislation, Definitions, and Assumptions

**Figure 2:**
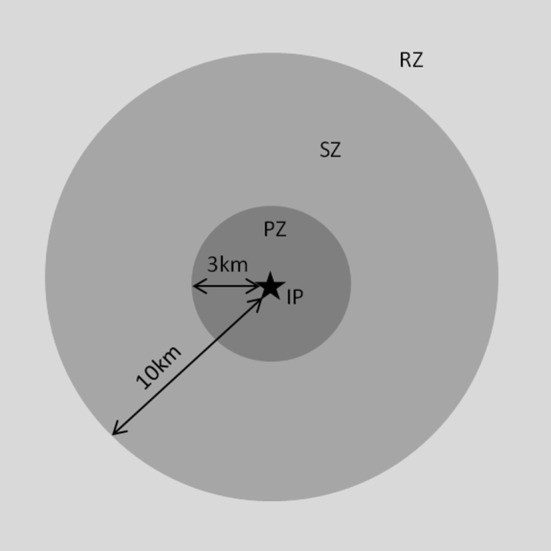
Zoning during an outbreak: Protection, surveillance and restricted zones. During an outbreak of FMD, movement control zones are put in place to help control spread of disease. A protection zone (PZ) is set up around an infected premises (IP), with a minimum radius of 3 km, or more if necessary to control disease. A surveillance zone (SZ), with a minimum radius of 10 km from the infected premises, is set up around the protection zone. A restricted zone (RZ) is set up outside these areas and extends as far as necessary to prevent disease spread; it may extend to the whole of Scotland. The number and extent of these zones changes as the outbreak progresses with new zones being created around newly identified infected premises. Zones are lifted as disease is eradicated from premises once disinfection and verification of disease freedom is met.

For the purposes of these VRAs, “access to the countryside” was defined as recreational activity involving people, vehicles, equipment, and in some cases, animals. Statutory disease control requirements limit access and activities on premises where FMD is suspected or confirmed (3). Control zones are put in place on suspicion and confirmation of disease to prevent spread of disease (Figure 2). When suspicion of disease cannot be ruled out, and diagnostic samples are taken from suspected cases, a Temporary Control Zone is put in place surrounding the suspect premises. On disease confirmation, a protection zone (PZ) is set up around an infected premises, with a minimum radius of 3 km, or more if necessary to control disease. A surveillance zone (SZ), with a minimum radius of 10 km from the infected premises, is set up around the protection zone. A national movement ban is enforced by introducing a Restricted Zone (RZ). These zones place restrictions on movements and activities around infected premises to prevent spread of disease. Later in the outbreak, restrictions may be relaxed either through reducing the size of the RZ or through allowing some resumption of normal activities under license within the RZ, SZ, or PZ. In this VRA, RZ is used to refer to areas which are within the RZ, but do not also fall within the PZ or SZ. Some rural activities are specifically prohibited within particular control zones, for example deer stalking and drag hunting are not permitted within a PZ. Land access rights within Scotland are liberal (21) although Scottish Ministers and local authorities have ability to restrict access for disease control purposes.

### Release and Exposure Assessment

Each step in the risk pathway is discussed below. A summary of key factors, uncertainties and likelihood levels for each step in the pathway is presented in Table 2. Mitigation measures specific to each activity are provided in the individual VRA documents (https://www.gov.scot/publications/foot-and-mouth-disease-veterinary-risk-assessments-vras/) and key mitigation measures are summarized in Table 2.

**Table 2:** Key factors, uncertainties and likelihood levels for each step of the risk pathway.

**Evidence for each step of risk pathway**	**Key knowledge gaps and uncertainties**	**Likelihood level without mitigation, key risk factors**	**Likelihood levels with mitigation, key mitigation measures**
**1. Risk of contamination from infection sources: Persons, animals, vehicles, or other equipment are contaminated when leaving homepremises**			
- Proximity to detected infected premises - Proximity to undetected infectedpremises - Outbreak stage - Virus strain - Livestock speciespresent - Degree of contact with livestock - Cleansing anddisinfection	Likelihood of virus survival on different materials and under different conditions Quantitative data on the likelihood of transmission via people, animals, vehicles, and equipment under differentconditions	PZ—low/medium SZ—low RZ and rest of country—very low Key factors influencingrisk: - Contact with susceptible livestock increases risk - Stage of outbreak—early in outbreak uncertainty regarding undetected infection increasesrisk	PZ—low SZ—very low RZ and rest of country—very low Key mitigationmeasures: - Cleansing and disinfection on leaving home premises - People/vehicles/equipment that have had contact with IP not to visit areas with susceptiblelivestock
**2. Risk of contamination from infection sources: Persons, animals, vehicles, or other equipment become contaminated on route to/from theactivity**			
- Proximity of route to premises with detected or undetected FMD - Length and duration of journey - Number and nature of stops - Cleansing anddisinfection	Quantitative data on the likelihood of transmission via people, animals, vehicles, and equipment under different conditions	PZ—low/medium SZ—low RZ and rest of country—very low Keyfactors: - Stage of outbreak—early in outbreak risk is higher - Stops at premises with or close to susceptible livestock, multiplestops	PZ—low SZ—very low RZ and rest of country—very low Key mitigationmeasures: - Cleansing and disinfection of vehicle on arriving at activity - Avoiding multiple stops, especially on equinepremises
**3. Risk of contamination from infection sources: Persons, animals, vehicles, or other equipment contaminate roads or environment and/or come into contact with susceptible livestock on route to/from theactivity**			
- Proximity of route to premises with susceptible livestock - Length and duration of journey - Number and nature ofstops	Quantitative data on the likelihood of transmission via people, animals, vehicles, and equipment under different conditions	Low Keyfactors: - Stops at premises with or close to susceptiblelivestock	Low Key mitigationmeasures: - Cleansing and disinfection on leaving home premises, and at any other stops - Avoiding multiple stops, especially on equinepremises
**4. Risk of contamination from infection sources: Persons, animals vehicles, or other equipment become contaminated via environment or contact with infected livestock duringactivity**			
- Proximity to premises with detected or undetected FMD - Presence and density of susceptible livestock at the location where the activity takes place - Presence of free ranging dogs - Level of use of land where activity takes place - Wildlife in locality - Meteorologicalconditions	Virus survival in different meteorological and ecological conditions	PZ—low to medium/high SZ—low to medium RZ and rest of country—very low tolow/medium Keyfactors: - Stage of outbreak—early in outbreak risk levels higher - Presence (current or recent) of susceptible livestock in area activity is taking place - Proximity, density, and likelihood of contact with susceptible species - Disturbance of wildlife - Number of peopleattending	PZ—low to medium SZ—low to low/medium RZ and rest of country—very low to low Key mitigationmeasures: - Preventing public coming into contact with livestock - Not parking vehicles where they can come into contact with livestock or feces - Keeping dogs onleads
**5. Risk of contamination from infection sources: Persons, animals, vehicles, or other equipment contaminate environment or new areas and/or come into contact with susceptible livestock duringactivity**			
- Presence and density of susceptible livestock at the location where the activity takes place - Number of people, animals, vehicles doingactivity	Quantitative data on the likelihood of transmission via people, animals, vehicles, and equipment under different conditions	Low to medium/high Keyfactors: - Presence (current or future) of susceptible livestock in area activity is taking place - Proximity, density, and likelihood of contact with susceptible species - Disturbance of wildlife - Number of peopleattending	Low Key mitigationmeasures: - Preventing public coming into contact with livestock, e.g., encourage public to stick to footpaths - Not parking vehicles where they can come into contact with livestock - Keeping dogs onleads
**6. Exposure and infection: Susceptible livestock come into contact with contaminated area, persons, animals, orvehicles**			
- Presence and density of susceptible livestock at the location where the activity takes place, or on contaminated routes - Meteorologicalconditions	Virus survival in different meteorological and ecological conditions	Very low to medium Keyfactors: - Proximity, density, and likelihood of contact of susceptible livestock - Presence of susceptible livestock at homepremises	Very low to medium Key mitigationsmeasures: - Keep susceptible livestock away from potentially contaminated areas (e.g., during and after events held on agricultural land) - Cleansing and disinfection on return to homepremises
**7. Exposure and infection: Susceptible livestock exposed to contamination becomeinfected**			
- Degree of contamination (viral dose) - Livestockspecies	No specific uncertainties	Very low to medium - Virus load present in contamination - Species	Very low to medium

*Ranges of likelihood levels are provided in column 3 and 4 to illustrate the likelihood levels for different activities The key factors that drive these likelihood levels (often factors that are specific to individual activities) are indicated*.

#### Release Assessment

##### Risk of contamination from infection sources: Persons, animals, vehicles, or other equipment are contaminated when leaving home premises

People, fomites or non-susceptible animals present a risk of FMD transmission if they become contaminated with FMDV. The likelihood of contamination is greatest on or close to premises with FMD. Premises with FMD may be detected (“infected premises”) or as yet undetected. On detected infected premises, control measures are in place to reduce the likelihood of FMDV contamination of people, fomites or the environment. However, contamination remains a possibility. In a PZ there are known infected premises, in a SZ known infected premises are located >3 km away and in a RZ known infected premises are located >10 km away (3). Once a national movement ban is in place, most transmission occurs by local spread (<3 km from a premises with FMD) (8, 22, 23), so zone is also a reasonable indicator of the likelihood of proximity to undetected infected premises. Early in the outbreak, there is increased risk of undetected infection in all zones. The risk of undetected premises with FMD arising from spread over longer distances can be better quantified by analysis of movement data to identify movements of animals from areas where FMD has been detected that have occurred before the implementation of movement restrictions. The likelihood of detection and transmission is also influenced by FMD virus strain.

FMD may be present on premises but remain undetected because (i) animals show no or mild clinical signs; (ii) animals are incubating infection; (iii) animals show clinical signs but these are not observed; or (iv) clinical signs are not reported. Although the peak of transmission occurs shortly after the appearance of clinical signs (24), infected livestock may excrete FMDV for several days before the appearance of clinical signs or in the absence of clinical signs, potentially leading to transmission or contamination prior to disease detection, particularly in cattle and pigs (25). Transmission via contaminated surfaces has been documented before the onset of clinical signs (26). FMD in sheep can be difficult to detect clinically as not all animals show clinical signs, and clinical signs are usually mild and short lived (27). There is therefore a greater risk of undetected infection on sheep-only premises.

*Contamination of people* People can carry FMDV on their clothes, footwear and bodies and pass it to susceptible animals. Veterinarians and other people were incriminated in spread leading to 10 of 51 outbreaks during the 1967–1968 outbreak in UK (28). When people handled pigs infected with FMDV then immediately handled susceptible sheep and pigs, all animals became infected (29). Including hand washing and changing outer wear reduced the risk on onward infection, whilst showering and changing outer wear prevented it (29, 30). It should be noted that these infections occurred when contact with susceptible animals immediately followed handling of pigs with clear signs of FMD, in laboratory conditions. The likelihood of similar transmission from handling animals that are incubating an infection, or that only show mild clinical signs, such as sheep, is much lower.

There is also evidence that people can carry FMDV in their nasal cavities, but the likelihood of this leading to infection in susceptible animals without close and prolonged contact is negligible. One incident is described where infection was passed from sick pigs, via people, to a susceptible cow, despite the people involved fully disinfecting, showering and changing clothes. The infection was assumed to have passed via the nasal cavity. However, this required prolonged contact with infected pigs and deliberate coughing, blowing and sneezing on the muzzles of the susceptible cattle (31). No FMD virus was detected in nasal swab samples collected from four investigators 12–84 h after they had been exposed to the virus, but virus was detected in the nasal swab from one of four investigators immediately after examining sick pigs at post-mortem (29). Screening of nasal swabs over several experiments following handling of various combinations of infected cattle, sheep, and pigs showed swabs to frequently test positive for virus by PCR but only occasionally on virus inoculation, and only one person tested positive the next day (PCR only), suggesting the likelihood of virus survival in the nasal cavities of personnel 16–22 h after exposure to infected animals is very small (32). Again, although theoretically possible, the likelihood of transmission via virus survival in a person's nasal cavity due to contact with animals on an undetected premises, or from animals that are not showing obvious clinical signs, is very small.

The risk of contamination is greatest if people have had contact with infected animals, and next greatest if they have had indirect contact, for example if they have been to premises where FMD is present (either detected or undetected) but not handled livestock. The risk is therefore present for anyone who keeps susceptible livestock or has visited premises with susceptible livestock (including occupational exposure such as veterinarians) due to the risk of undetected infection. The likelihood and amount of contamination varies with species infected, stage of infection, degree of contact, and any biosecurity measures in place (29). Although it is known that contaminated people have played a role in causing new outbreaks (8, 33, 34), there is not sufficient information to quantify the risk with any certainty.

*Contamination of vehicles and equipment*. Vehicles and equipment can act as fomites. Virus can survive in slurry for up to 9 days at 20°C, to 14 weeks at 5°C (35). Virus is also still viable when dried onto surfaces (36, 37), although the length of time virus can survive for is less well-quantified. Contaminated vehicles and equipment have been implicated in spread in previous outbreaks (8, 28, 33). In UK in 2007, vehicles contaminated with virus from a laboratory effluent system were suspected to have moved virus to susceptible cattle farms (38). As with contamination of people, the greatest risks of contamination are associated with vehicles that have visited premises where FMD is present, which is most likely to occur if they are located close to areas where FMD is known to be present (i.e., the PZ or SZ). Keeping susceptible livestock or visiting premises with susceptible livestock also present an increased risk due to the possibility of undetected infection. Equipment may be contaminated from use in other areas, for example boats, bicycles, riding equipment, fishing tackle, guns. However, there is not sufficient quantitative information to assess the risk with any certainty.

*Contamination of non-susceptible animals such as dogs and horses*. Animals such as dogs and horses are not susceptible to FMD but may become contaminated and carry the virus mechanically (12). The likelihood of dogs being contaminated, and the factors that influence it, are similar as for people and will be greatest if dogs have had access to infected livestock or livestock products (34). Equine premises are often close to or associated with premises where susceptible livestock are kept and may source products such as straw from livestock-keeping premises, which can act as fomites. FMDV can survive on bedding and food stuffs such as hay, straw and bran for over 100 days at 16°C [reviewed by (1)] or longer in winter [reviewed by (2)]. Imported straw was identified as the most likely incursion route for an outbreak in Japan in 2000 (39). The proximity of the equine premises to areas where FMD is known to be present, movement history of horses, presence of susceptible livestock on the same or adjacent premises, and sources of feed and bedding will influence the risk that horses are contaminated. Events that bring together large numbers of horses from multiple areas, such as events, point to points, shows, competitions, drag hunts, and race meets present an increased risk of contaminated horses being present.

##### Risk of contamination from infection sources: Persons, animals, vehicles, or other equipment become contaminated on the route to/from the activity

People, vehicles or other equipment could become contaminated whilst traveling to the location of the activity. The likelihood depends on the proximity of the route to known infected premises, the presence of undetected infected premises (which is influenced by the stage of the outbreak and zone as discussed above), the length of the journey and the number of stops or destinations. Any stops made at premises where susceptible livestock are kept, or are close by, increase the likelihood of contamination. For example, horseboxes traveling to events where they stop at multiple yards to collect horses would have a higher likelihood of becoming contaminated on the route, as well as increasing the risk of moving FMDV between premises. The amount of farm traffic and animal movements, and biosecurity arrangements of local premises will influence the likelihood that roads are contaminated.

##### Risk of contamination from infection sources: Persons, animals, vehicles, or other equipment contaminate roads or environment and/or come into contact with susceptible livestock on route to/from the activity

Contaminated people, vehicles or other equipment may contaminate the roads or surrounding areas whilst traveling to the location of the activity. As for step 2, this risk is influenced by the length of the journey and the number of stops made, particularly on premises where susceptible livestock are kept, or are close by. The likelihood of coming into contact with susceptible livestock is influenced by the density and proximity of livestock in the area.

Whilst a contaminated person, animal or vehicle could lead to further contamination, the virus load by this stage would be very low.

##### Risk of contamination from infection sources: Persons, animals, vehicles, or other equipment become contaminated via environment or contact with infected livestock during activity

People, vehicles, equipment, or non-susceptible species such as dogs or horses may come into contact with contaminated areas or infected livestock during the activity. Areas may become contaminated from current or previous presence of infected livestock or livestock products, from infected wildlife, or through movement of contamination on fomites. FMDV-infected livestock may be detected (in which case by the premises is bound by statutory regulations and entry is not permitted) or undetected. Contamination of areas used for recreational activities by infected livestock, or risk of contact with infected livestock, is therefore likely to be due to undetected infection. This is most likely to happen close to infected premises, or at the start of an outbreak when the risk of widely dispersed undetected premises is higher due to animal movements (see step 1 for detailed consideration of premises with undetected infection). The likelihood that the presence of infected animals on the premises leads to contamination of areas where activities might take place, depends on how recently the infected animals were present, number of infected animals, species, virus excretion, environment conditions (which influence FMDV survival), and management and grazing patterns (which vary by season). Contamination could also be introduced via muck spreading with contaminated feces or other by-products.

In other parts of the world, wildlife can play an important role in FMD transmission (40). All British deer species are susceptible to infection and can transmit virus to domestic livestock experimentally (41). Wild boar are susceptible and can transmit infection to domestic pigs, although boar show only mild clinical signs (42). Sero-surveys and diagnostic testing of deer and wild boar did not reveal any positive animals in UK, Netherlands or Germany following the outbreaks in 2001 in livestock (43, 44). However, seropositive roe deer and wild boar were found following livestock outbreaks in Thrace (45, 46). In Europe, models usually conclude that deer and boar populations cannot maintain infection in the absence of outbreaks in livestock (45, 47). Although there is no evidence that deer or boar have played a role in FMD transmission in UK, their involvement in facilitating local disease spread does remain a risk.

Other species such as hedgehogs and rodents can be infected with FMDV, but are unlikely to be very important in transmission (48, 49). Wildlife can also move FMDV mechanically if they become contaminated, for example scavengers such as seagulls, crows and foxes (33, 34, 50). Overall, the risks of further spread of FMDV associated with wildlife are very low but any activity which causes disturbance to wildlife does increase this risk, especially close to premises where FMD is present.

##### Risk of contamination from infection sources: Persons, animals, vehicles, or other equipment contaminate environment or new areas and/or come into contact with susceptible livestock during activity

Contaminated people, vehicles or equipment, or non-susceptible species such as horses or dogs, may introduce contamination to the area. The potential area that could be contaminated is related to the type of activity. The likelihood also varies with the number of people, vehicles, equipment, and non-susceptible species involved, the number that are contaminated and the degree of contamination. Dogs or horses may spread contamination over larger areas than, for example, walkers alone. The proximity, density, and the management type of any susceptible livestock influences the likelihood of contact, for example for penned dairy cows would be feasible to prevent contact with people and dogs but this could be difficult for extensive sheep production.

If people, vehicles, equipment, or non-susceptible species such as dogs or horses become contaminated during the activity they may move contamination into new areas. This is particularly important for activities that involve more than one premises, for example drag hunting that may cover land associated with multiple premises. The risks are influenced by the distance traveled or area covered, number of premises involved, and the number of people, vehicles, equipment, and non-susceptible species involved.

Any potential contact between contaminated people, vehicles or other equipment, or non-susceptible species such as horses or dogs, and susceptible livestock presents a risk of transmission. The greatest risks are associated with the presence of susceptible livestock in the area where the activity is taking place. The likelihood of contaminated people, vehicles or other equipment coming into contact with susceptible livestock during the activity also depends on the type of activity being conducted, the area or distance covered during the activity, and the type of land used for the activity. If the activity is taking place in areas which are not agricultural land and are never used for grazing susceptible livestock or growing feed or bedding for susceptible livestock, the risks are negligible. Activities that involve greater numbers of people increase the risk that some will be contaminated. If the number of contaminated personnel and vehicles is greater, the total probable amount of FMDV that would be released would increase.

Dogs, if present for example for walking, or for deer stalking or shooting birds, may cover larger distances and be more likely to come into contact with susceptible livestock. In addition, the presence of dogs can encourage cattle to approach and may increase the risk of transmission, if dogs or people are contaminated. It is possible that contaminated people, vehicles, equipment, or dogs could come into contact with susceptible wildlife. Whilst any contact between people and deer is only likely to occur with deer that have been shot, and are therefore being removed, susceptible species such as deer could come into contact with contaminated vehicles. Dogs (particularly if not restrained) or horses may also disturb wildlife, increasing the risk of virus dissemination by infected wildlife, as is also the case for any events that could lead to movement of wildlife such as deer stalking.

#### Exposure Assessment

##### Exposure and infection: Susceptible livestock come into contact with contaminated area, persons, animals, or vehicles

Susceptible livestock could come into contact with contamination left on the route, during the activity or if contaminated people return to their home premises where livestock are kept. There are also risks for livestock which are later moved onto to an area where contamination has been introduced due to survival of FMDV in the environment. FMDV can survive on soil for 2–5 days at temperatures above 16°C, up to 5 weeks at 3–7.5°C, and over 20 weeks under snow or at temperatures below 5°C [reviewed by (1, 2)]. Survival duration increases with decreasing temperatures, increasing relative humidity and presence of organic material and varies with virus strain [reviewed by (1)]. There are therefore risks to livestock that come into contact with an area where contamination has been introduced, even after some time as passed, in the right conditions. The likelihood of this happening is influenced by the presence, proximity and density of susceptible livestock in the contaminated area, and degree of contamination.

If roads are contaminated, susceptible livestock could come into contact with FMDV, if (i) livestock are moved on public roads; (ii) livestock adjacent to public roads are exposed; or (iii) contamination is moved, for example on other vehicles, into premises where susceptible livestock are kept. In the PZ, SZ, and RZ, movements of livestock on public roads are not permitted except under license for specific activities, for example movement of dairy animals for milking.

##### Exposure and infection: Susceptible livestock exposed to contamination become infected

If susceptible livestock are exposed to FMDV they may become infected. The likelihood that exposure of susceptible livestock to FMDV in the environment results in infection is not well-characterized (51), but is likely to vary by species, the virus dose exposed to and transmission route. When considering the infection of susceptible livestock via contaminated environment, the transmission route could be aerosol or oral. Cattle and sheep are relatively susceptible to aerosol infection, whilst pigs are not (52–54). Pigs are more susceptible than ruminants by the oral route, although higher doses are generally require for oral infection than aerosol infection [reviewed by (13)]. In general transmissibility is moderate when animals are kept in close contact; not all exposed animals become infected, and some only sub-clinically (13, 55). The variability observed through experimental infections suggests transmission would be much less efficient when animals are outdoors and in less close contact (56, 57).

The likelihood of infection is proportional to the virus dose (53). Indirect transmission is likely to involve lower virus doses than direct transmission and therefore is less likely to result in infection. The likelihood of infection occurring following fomite to fomite transmission can therefore be assumed to be very low.

### Consequence Assessment

The end point of the risk pathway for all activities was the presence of infected livestock on a previously uninfected premises. The consequences of this include the health and economic impacts both for the individual farm infected, and for the wider livestock industry and economy, of prolonging an FMD outbreak. Since the consequences for each risk pathway were the same, only the likelihood levels are presented here.

Final likelihood levels for each activity in the PZ, SZ and RZ are presented in Table 3.

**Table 3:** Likelihood levels of the activities assessed in the protection zone, surveillance zone, and restricted zone.

**Activity**	**Protection zone**	**Surveillance zone**	**Restricted zone**
Walking	Medium^a^	Medium	Low
	*Medium*	*Low*	*Very low*
Cycling	Medium	Medium	Low
	*Medium*	*Low*	*Very low*
Canoeing	Medium	Medium	Low
	*Medium*	*Low*	*Very low*
Fishing	Medium	Medium	Low
	*Medium*	*Low*	*Very low*
Horse riding	Medium	Medium	Low
	*Medium*	*Low/medium*	*Very low*
Staging an equestrian event on agricultural land	Medium/high	Medium	Low/medium
	*Medium*	*Low/medium*	*Low*
Staging a race meet	Medium/high	Medium	Low
	*Medium*	*Low/medium*	*Low*
Staging other events on agricultural land	Medium	Medium	Low
	*Medium*	*Low*	*Very low*
Organized sporting events	Medium	Medium	Low
	*Medium*	*Low/medium*	*Low*
Drag hunting	Not permitted	Medium	Low
	*Not permitted*	*Medium*	*Very low*
Stalking/shooting deer	Not permitted	Medium	Low/medium
	*Not permitted*	*Medium*	*Low*
Shooting birds	Medium	Medium	Low
	*Medium*	*Low*	*Very low*

a *Likelihood levels are shown without any mitigation strategies in place (not italics), and assuming mitigation strategies are in place and complied with (italics)*.

## Discussion

The movement of people (and other non-susceptible animals) to, from and during activities in the countryside during an FMD outbreak carries a risk of indirect spread of FMDV to uninfected farms. Indirect transmission of FMDV via fomites is an important potential source of infection, and any vehicles, people, non-susceptible animals or equipment that come into contact with FMDV present a risk of passing disease to any livestock with which they subsequently come into contact. This study assessed the risks associated with access to the countryside during an FMD outbreak to allow increased transparency in future decision-making.

For most activities, the likelihood of causing new outbreaks of FMD was assessed to be very low in the RZ, assuming compliance with specified mitigation strategies. Risk increases in the SZ and is greatest in the PZ, closest to identified infected premises. This is predominantly due to the risks associated with local spread leading to contamination or the roads and environment close to known infected premises, and the increased risk of undetected infected premises. In the early stages of an outbreak, the likelihood of undetected infected premises is greatest and the geographical extent of the outbreak is most uncertain, hence a more conservative assessment of risk is appropriate during this period. Across all zones, activities which increase the risk level are those that involve large groups of people, vehicles, movement of non-susceptible animals such as dogs and horses, and activities involving susceptible wildlife species. Although horses are not susceptible to FMD, a sizeable proportion of horse stables are closely associated with other livestock enterprises in various ways (e.g., sheep grazing on nearby premises). Therefore, horses (and associated people, vehicles, and equipment) are considered a more likely vehicle for FMDV in the face of an outbreak than other non-susceptible animals or people from the general public. An additional factor in Scotland is that countryside access is not limited to paths or specific areas (21), and there are likely to be more opportunities for people to come into contact with livestock, wildlife and contaminated areas.

The conclusion of the VRAs was that for most activities, the likelihood of causing new outbreaks of FMD is considered to be medium (occurs regularly) in the PZ, low (rare but could occur) in the SZ, and very low (very rare but cannot be excluded) in the RZ, assuming compliance with specified mitigation strategies (Table 2). However, the likelihood of new outbreaks associated with hunting, shooting, stalking, and equestrian activities is considered to be greater.

The most important source of uncertainty within the VRAs is attributable to a paucity of data on the likelihood of transmission via fomites. The lack of data is perhaps surprising given the priority given to FMD research since 2001. However, meaningful field data on fomite transmission would be virtually impossible to collect during an outbreak and experimental studies are expensive to conduct and could only ever partially address this question.

There are three main options for management of the risks associated with countryside access: (i) do not permit access to the countryside; (ii) do not permit activities when or where the risk of FMDV being present is greatest (i.e., in a PZ or SZ, in early stages of an outbreak, or over agricultural land where susceptible livestock are present); (iii) permit activities from the early stages of an outbreak but under certain conditions. This study highlights that there is no justification for automatically preventing access to the countryside at a Scottish level. Real risks remain, particularly close to premises with FMD, but for most activities, the risk is very low at greater distances from premises with FMD, particularly once the early stage of an outbreak have passed and the likelihood of undetected infection has reduced. Therefore, options (ii) or (iii) are appropriate, depending on the activity. Specific mitigation measures are listed in the individual VRA documents.

Much of the information available to inform this analysis is based on FMDV serotype O. This serotype has been responsible for most of the large outbreaks that have occurred in countries that are usually FMD-free in the last few decades, and many of the reported experimental studies on transmission and survival of FMDV use serotype O [for example (24, 25, 29, 35)]. However, incursions of other serotypes could occur. Species susceptibility, length of incubation period, ease of detecting clinical signs and transmission are known to vary between serotypes and strains. For example, pigs infected with serotype C produced more aerosol virus that those infected with serotype O (17), while pigs infected with serotype A shed more virus than pigs infected with O or Asia (18). Whilst there is no clear evidence that virus survival in the environment, likelihood of infection via fomites, or susceptibility of wildlife species differ between serotypes, these aspects have not been widely studied and there is not sufficient information available to have confidence that differences do not exist. Any differences to these parameters could affect the risk levels described.

Although this risk assessment focused on Scotland, the risk pathways described here are likely to be appropriate for other countries that are usually FMD free without vaccination. However, the factors that influence the risk level at each step of the pathway may vary between countries, depending on factors such as the likelihood of undetected infected premises, the likelihood of coming into contact with livestock on the way to/from or whilst conducting the activity (which could depend on the specific nature of outdoor activities and the ways that susceptible livestock are kept) and the likelihood that susceptible wildlife are present. These risk factors and the subsequent likelihood levels should be reviewed if the risk assessments are to be used elsewhere.

A risk assessment approach to this issue was appropriate because it allowed the available evidence to be compiled and assessed, whilst still provided documents that can be used by policy-makers for decision making. A qualitative approach was taken in this study because there were insufficient data to support a quantitative approach. A risk matrix approach, widely used including in veterinary science (58) was considered. However, risk matrices can give a false impression of scientific robustness, whilst actually relying on subjective risk level estimates which may be influenced by a range of other considerations such as personal knowledge and beliefs (59, 60). Examples of uses within the field of veterinary medicine have also highlighted the issues of the inability to account for marked variation in estimates within categories, and loss of information with successive levels of coding, particularly when the model does not take a modular step-wise form (61). Therefore, we used a qualitative descriptive approach that would allow us to conclude an overall risk level and highlight areas of particular uncertainty and variability. It is acknowledged that formally soliciting expert opinion (62) may have reduced uncertainty in some parameters. However, the policy imperative did not allow time for this and the subsequent rounds of peer-review process reassured us that our original risk estimates would not be substantially altered.

## Data Availability Statement

The datasets generated for this study are available on request to the corresponding author.

## Author Contributions

HA, LB, DM, and GG conceived and designed the study. HA and LB performed the analysis. HA, LB, and DM wrote sections of the manuscript. All authors contributed to manuscript revision, read, and approved the submitted version.

### Conflict of Interest

The authors declare that the research was conducted in the absence of any commercial or financial relationships that could be construed as a potential conflict of interest.
